# Direct Observation of Enhanced Raman Scattering on Nano-Sized ZrO_2_ Substrate: Charge-Transfer Contribution

**DOI:** 10.3389/fchem.2019.00245

**Published:** 2019-04-17

**Authors:** Peng Ji, Zhe Wang, Xiaohong Shang, Yu Zhang, Yikuan Liu, Zhu Mao, Xiumin Shi

**Affiliations:** ^1^School of Chemistry and Life Science, Changchun University of Technology, Changchun, China; ^2^College of Chemical Engineering, Changchun University of Technology, Changchun, China

**Keywords:** raman, SERS, ZrO_2_, charge-transfer, nanoparticle

## Abstract

Direct observation of the surface-enhanced Raman scattering (SERS) of molecules adsorbed on nano-sized zirconia (ZrO_2_) substrates was first reported without the need for the addition of metal particles. It was found that ZrO_2_ nanoparticles can exhibit unprecedented Raman signal enhancements on the order of 10^3^ for the probe molecule 4-mercaptobenzoic acid (4-MBA). The dramatic effect of the calcination temperature on the ZrO_2_ nanoparticles was also investigated. The ZrO_2_ nanoparticles with the particle diameter of 10.5 nm, which were prepared by calcination at a temperature of 500°C, have the highest SERS activity. A comparison between the experimental and calculation results indicates that charge transfer (CT) effects dominate the surface enhancement. The plentiful surface state of ZrO_2_ active substrate that is beneficial to CT resonance occurs between molecules and ZrO_2_ to produce a SERS effect. The CT process depends, to a large extent, on the intrinsic properties of the modifying molecules and the surface properties of the ZrO_2_. This is a new SERS phenomenon for ZrO_2_ that will expand the application of ZrO_2_ to microanalysis and is beneficial for studying the basic properties of both ZrO_2_ and SERS.

## Introduction

The large surface-enhanced Raman scattering (SERS) enhancement observed in metals and semiconductors is explained by the vibrational coupling of various resonances in the molecules of the semiconductor system, such as surface plasmons, excitons, charge transfer (CT), and molecular resonance (Yin et al., 2010; Li et al., 2011; Wang and Chen, 2011; Jiang et al., 2013; Wang et al., 2013, 2014, 2016; Huang et al., 2014; Kadkhodazadeh et al., 2014; Mogensen and Kneipp, 2014; Shiohara et al., 2014; Zhao et al., 2014; Shen et al., 2015; Scott and Carron, 2016; Fu et al., 2019). Because semiconductor materials are widely used in the rapidly developing energy and optoelectronic fields, it is important to investigate the SERS mechanism for semiconductor substrates (Lombardi and Birke, 2014; Alessandri and Lombardi, 2016; Han et al., 2017). Semiconductors that can be used as SERS substrates have been identified, such as TiO_2_, ZnO, and CdS. These can amplify the Raman signals of surface-adsorbed organic molecules such as 4-mercaptopyridine (4-MPY) and 4-mercaptobenzoic acid (4-MBA) by more than 100-fold (Sun et al., 2007; Wang et al., 2007; Yang et al., 2008).

Nano-sized zirconium dioxide (ZrO_2_) is one of the most important materials for the preparation of nano-ceramics, and it can prepare ceramic components with various functions (Li et al., 2017). It has applications and developments in solid oxide fuel cells, thermal barrier coating materials, catalyst carriers, lubricant additives, medical, gas sensitive, and wear resistant materials (Duan et al., 2017; Gan et al., 2017; Long et al., 2017; Xu et al., 2017; Wang et al., 2019). The brittleness of ceramic materials limits its practical application, and nanoceramics are a strategic way to solve the brittleness of ceramics. However, grain size is the most important factor affecting the performance of nano-ceramics. The decrease of grain size will significantly improve the mechanical properties of materials (Zhang L. et al., 2015). The grain refinement will contribute to the slip between the grains, giving the material plastic behavior. A large number of studies by researchers have shown that the temperature is closely related to the size of ZrO_2_ particles, and the transition temperature of ZrO_2_ particles below 100 nm can be greatly reduced (Becker et al., 2008). Toughening of ceramics by microcracks and residual stresses generated the conversion of ZrO_2_ from a tetragonal phase (t-ZrO_2_) to a monoclinic phase (m-ZrO_2_). Therefore, the nano-sized ZrO_2_ can significantly increase the strength and stress intensity factor of the ceramic, thereby increasing the toughness of the ceramic (De Aza et al., 2002). Nowadays, the application research on nano-sized ZrO_2_ has become one of the research hotspots of nano-ceramic new technology. As a new type of high-performance ceramics, the use of highly sensitive SERS technology with *in-situ* characterization advantages to study the size effect of nano-sized ZrO_2_ is expected to promote the better performance of nano-ceramic materials to enable the emergence of new properties and functions.

**Figure 1:**
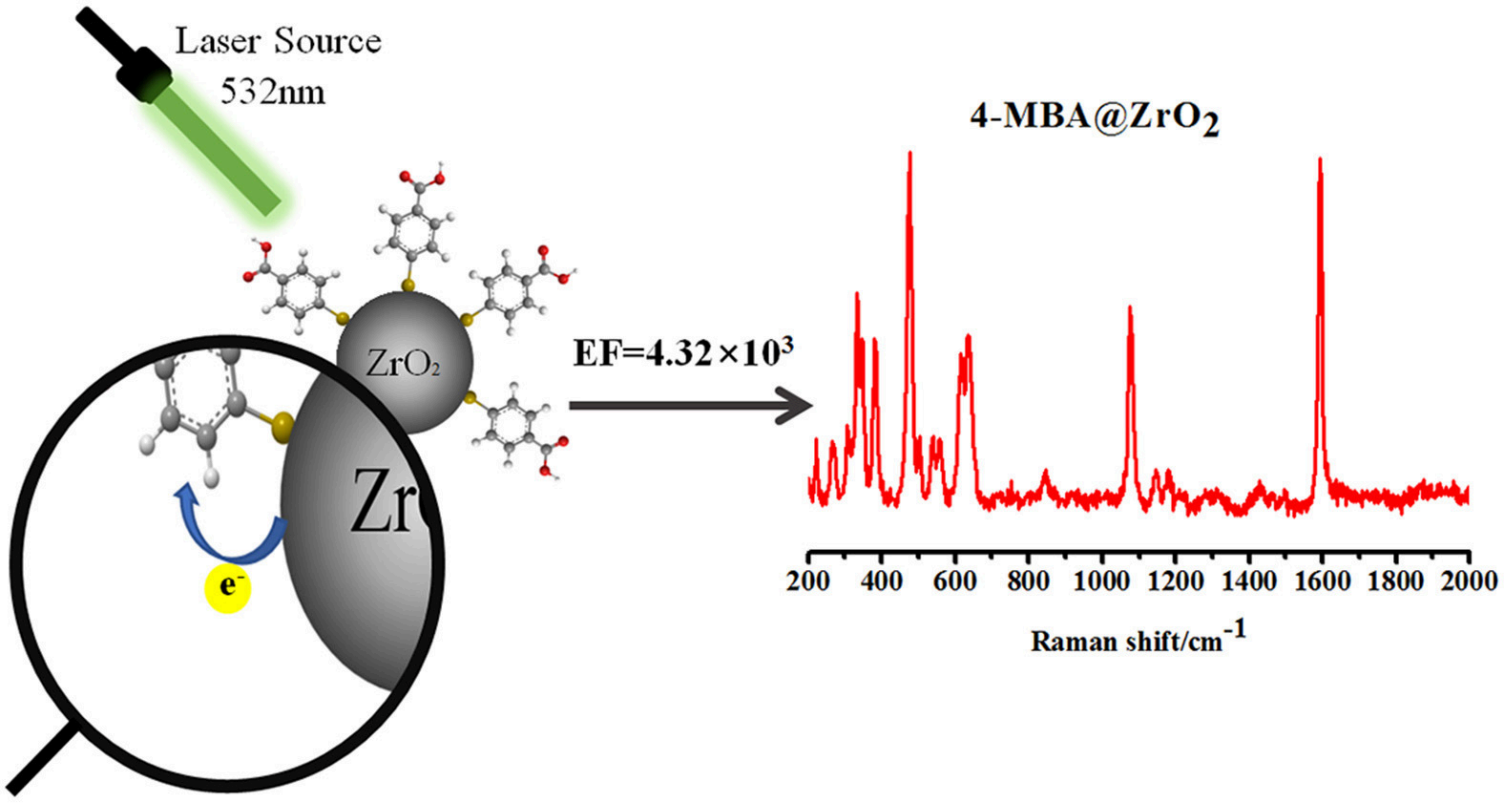
Schematic representation of ZrO_2_ nanoparticles with the organic molecule 4-MBA.

In addition, ZrO_2_ is a particularly attractive candidate to promote CT enhancement because it has recently been shown that the luminescence effect of ZrO_2_ materials is derived from transitions between new and ground state energy levels formed by oxygen vacancies that capture electrons and oxygen vacancies produced by the formed association center (Kralik et al., 1998; Chang and Doong, 2007). Owing to the photoelectric properties of ZrO_2_, a CT state is readily formed. Currently, there are no reports on the use of ZrO_2_ enhancement to obtain SERS (Figure 1).

## Materials and Methods

### Chemicals

Zirconium nitrate pentahydrate was obtained from Tianjin Guangfu Fine Chemical Research Institute; triethylamine was obtained from XiLong Chemical Co., Ltd.; CTAB were obtained from China Huishi Biochemical Reagent Co., Ltd. 4-Mercaptobenzoic acid (4-MBA), 4-Mercaptopyridine (4-Mpy), and P-aminothiophenol (PATP) were purchased from Sigma-Aldrich. The other chemicals are analytically pure without further purification.

### Synthesis of ZrO_2_ Nanoparticles

The synthesis of ZrO_2_ was followed the procedure below (Zhou et al., 2006). First, at room temperature and in a 500 mL round-bottomed flask, mix and dissolve 17.44 g Zirconium Nitrate and 2.95 g Hexadecyl trimethyl ammonium Bromide in 180 mL deionized water. The hydrolysis was carried out with slight stirring. Stirring was continued for about 20 min until a clear sol was obtained. 22.615 mL Triethylamine was added in the solution under rough stirring. The mixture was kept at 75°C for 8 h in the round-bottomed flask. The sol-gel production was dried at 82°C for 48 h. This was the last step after drying the product, eluted with ethanol for 48 h in a Soxhlet extraction. The product was calcined in a muffle furnace for 2 h to obtain ZrO_2_ nanoparticles at different temperatures (450–650°C).

### Adsorption of Probing Molecules

The surface modification of ZrO_2_ nanocrystals is obtained as follows: 10 mL 4-MBA ethanol solution concentration 1 × 10^−3^ M was used to dissolve 20 mg of ZrO_2_ nanocrystals. The mixture was stirred at room temperature for 4 h and then centrifuged. The 4-MBA molecules that did not adsorb were subsequently removed by rinsing the precipitate with absolute deionized water and ethanol once more. ZrO_2_ Nanoparticles that modify by 4-MBA and PATP are similar to 4-MBA.

### Sample Characterization

The crystal structure of the ZrO_2_ sample was determined by X-ray diffraction using a Rikagu Smartlab X-ray powder diffractometer with 0.15406 nm. The electronic absorption spectra were recorded on a Cary5000 UV-vis spectrophotometer. Raman spectra were obtained with Horiba HR Evolution Raman spectroscopy. The 532 nm radiation from an air-cooled argon ion laser was used as the exciting source. The morphology of ZrO_2_ nanoparticles was characterized by the JEM-2000EX TEM instrument. A Thermofisher Escalab 250XI X-ray photoelectron spectroscopy (XPS) was applied to investigate the elemental composition and the surface electronic valence state for UPS.

## Results and Discussion

### ZrO_2_ Phonon Modes (XRD & Raman)

Zirconia has three low-pressure structural phases. With increasing temperatures, zirconia changes from a monoclinic phase (m-ZrO_2_) to a tetragonal phase (t-ZrO_2_), eventually becoming a cubic phase (Zheng et al., 2009). The monoclinic phase (space group C5 2 h or P21/c) is thermodynamically stable at temperatures of < 1400 K. At around 1400 K, the t-ZrO_2_ transition (space group D15 4 h or P42/nmc) is stable until 2570 K, and is a slightly distorted form of the cubic structure (Heuer et al., 1982). In this study, the size of the ZrO_2_ nanoparticles was controlled by adjusting the calcination temperature to change the surface defect state of the nanoparticles to obtain a stronger SERS signal of the surface substance. This is verified by UV-vis spectra of ZrO_2_ nanoparticles with different calcination temperature, see Figure S1.

#### Increase in Nanoparticle Size With Increasing Calcination Temperature

**Figure 2:**
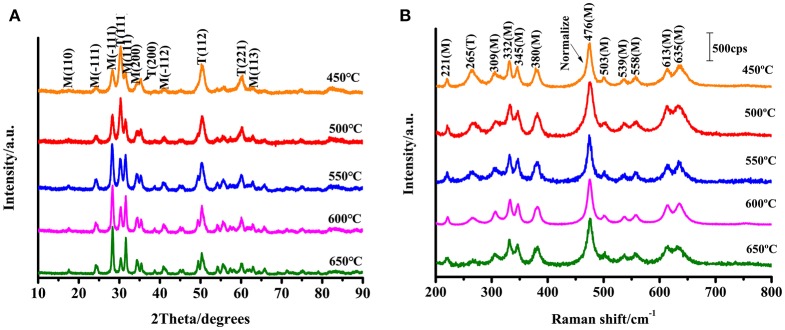
**(A)** XRD patterns of ZrO_2_ nanoparticles with different calcination temperature; **(B)** Raman spectra of ZrO_2_ with different calcination temperature with excitation 532 nm.

Figure 2A shows the XRD patterns of ZrO_2_ nanoparticles calcined at different temperatures. The m-ZrO_2_ is the dominant phase in these ZrO_2_ nanoparticles. Almost all of the diffraction peaks are comparable to the standard data and are remarkably sharp, indicating the purity and high crystallinity of the synthesized ZrO_2_ material. It can be observed that as the calcination temperature increases from 450 to 650°C, the XRD pattern becomes narrower and the sample crystallinity increases. The diameters of the ZrO_2_ nanoparticles calcined at 450, 500, 550, 600, and 650°C were calculated from the full width at half-maximum using the Scherrer equation. The calculated diameters of the zirconia nanoparticles were 8.1, 10.5, 11.1, 15.5, and 17.6 nm, respectively. Thus, it can be seen that the grain size increases with increasing calcination temperature. The wide XRD peak is attributed to a very small particle size, which is also demonstrated by the transmission electron microscopy (TEM) results (Figure S2). As the calcination temperature increases from 450 to 650°C, both of the crystallinity and grain size of the ZrO_2_ sample gradually increases. As shown in Figure S3, the XRD patterns show that the ZrO_2_ nanoparticles calcined at 500°C primarily exhibit m-ZrO_2_ (PDF card: 13-307), and there is extremely little t-ZrO_2_ present (PDF card: 88-1007).

#### Nanoparticles Primarily Exist in the m-Phase

ZrO_2_ samples were prepared through the sol-gel method and characterized using Raman spectroscopy (Figure 2B). The Raman shift characteristics reflect the energy level change in the specific vibration of the molecules. Tetragonal zirconia has six active vibrational modes (see Equation 1): B1g, Eg, B1g, Eg, A1g, and Eg (Zhao and Vanderbilt, 2002). Monoclinic zirconia has 18 Raman-active vibrational modes (see Equation 2).

(1) $$\Gamma_{\text{vib}}{}^{\text{t}}=\text{A1g}+\text{2B1g}+\text{3Eg}$$

(2) $$\Gamma_{\text{vib}}{}^{\text{m}}=\text{9Ag}+\text{9Bg}$$

From the Raman spectra and scattering data (see Figure 2B), it can be seen that the composition of the synthesized ZrO_2_ is a mixture of t-ZrO_2_ and m-ZrO_2_, with a greater proportion of monoclinic phase. Comparing the Raman spectra, it is observed that the prepared ZrO_2_ nanoparticles had an Eg vibrational mode at 265 cm^−1^ corresponding to the tetragonal phase. The remaining peaks occurred at 221 cm^−1^ (Bg), 309 cm^−1^ (Bg), 332 cm^−1^ (Bg), 345 cm^−1^ (Ag), 380 cm^−1^ (Ag), 476 cm^−1^ (Ag), 503 cm^−1^ (Bg), 539 cm^−1^ (Bg), 558 cm^−1^ (Ag), 613 cm^−1^ (Bg), and 635 cm^−1^ (Ag) are the vibration modes of m-ZrO_2_ (Carlone, 1992). Figure 2B shows that the 11 active vibrational modes correspond well with the monoclinic phase. This indicates that m-ZrO_2_ is dominant and the crystallization is relatively good. This point is consistent with the results observed using XRD; however, compared to the XRD patterns, the Raman spectra can reflect the difference in the crystallinity of ZrO_2_ caused by different calcination temperatures with greater sensitivity.

### Size-Dependent of SERS

**Figure 3:**
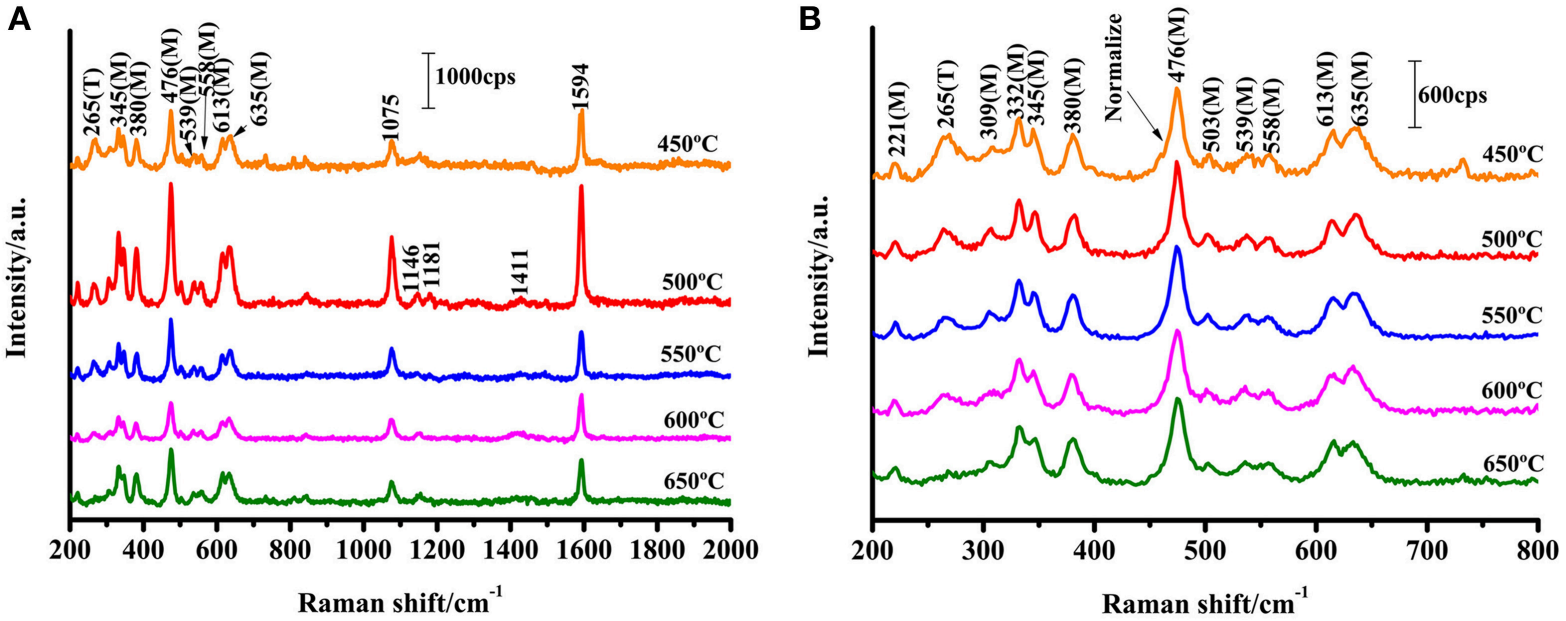
SERS spectra of 4-MBA adsorbed on ZrO_2_ nanoparticles with different calcination temperature with 532 nm excitation. **(B)** Is a magnification of **(A)** in the range of 200-800 cm^−1^.

ZrO_2_ is an n-type semiconductor, and thus the surface of ZrO_2_ nanoparticles are rich in oxygen vacancy defects (Navio et al., 2001). To study the effect of the calcination temperature of ZrO_2_ on the SERS enhancement, SERS spectra of 4-MBA adsorbed on ZrO_2_ nanoparticles calcined at different temperatures were obtained, as shown in Figure 3. The Raman spectra obtained for ZrO_2_ nanoparticles at varying calcination temperatures can be used to explore the role of surface defects on the CT mechanism. For calcination temperatures in excess of 500°C, as the zirconia calcination temperature increases, the crystallinity also increases, resulting in a decrease in the surface defect content of the ZrO_2_ nanoparticles (Figure 2A). This is also the reason for the decrease in CT effects of the ZrO_2_ molecules. Therefore, it can be seen from Figure 3 that at temperatures above 500°C, the SERS signal of zirconia decreases with increasing temperatures. This indicates that in the process of ZrO_2_ molecular CT, the surface defects play an important role and a rich surface state favors the ZrO_2_-to-molecule CT process.

The structural characteristics of the ZrO_2_ nanostructures are analyzed by TEM techniques, as shown in Figure S2. Based on the results, the crystalline sizes of all of the nanoparticles are ≈10 nm in diameter, which is similar to the results obtained from the XRD patterns through calculation with the Scherrer equation. The results indicate that the size of the ZrO_2_ nanoparticles increases with increasing calcination temperature. This indicates that the calcination temperature has a significant effect on the size of the nanoparticles. The optical properties of the ZrO_2_ nanoparticles were further analyzed by means of UV-Vis spectra.

### Calculation of the Surface Enhancement Factor (EF)

The surface enhancement factor (EF) was calculated for the 4-MBA adsorbed on ZrO_2_ nanoparticles at an excitation of 532 nm. The assignment of each of the strong peaks in the Raman spectrum is listed in Table S1. The ν (C-C) ring breathing mode (~1,594 cm^−1^) was selected to calculate the EF of ZrO_2_ nanoparticles. The EF calculation formula is shown in Equation (3):

(3) $$\text{EF}=\frac{I_{surf}N_{Bulk}}{I_{Bulk}N_{Surf}}$$

where *I* represents the corresponding intensity of the selected peak, and *N* represents the number of molecules observed by the laser spot; *N*_*Bulk*_ is the number of bulk 4-MBA molecules under laser irradiation, *N*_*Surf*_ is the number of 4-MBA molecules involved in the surface modification of ZrO_2_, *I*_*Surf*_is the intensity of a vibrational mode in the SERS, and *I*_*Bulk*_ is the intensity in the conventional Raman spectra of the same mode. From the area of the laser spot (~1.3 μm^2^) and the focus penetration depth of the laser (~10 μm), the volume of the solid sample can be calculated. Based on the density (1.5 g·cm^−3^) and volume of 4-MBA, *N*_*Bulk*_ was calculated to be 7.61 × 10^10^. Assuming that a single layer of ZrO_2_ nanoparticles are uniformly covered with a square centimeter of sediment, the number density is estimated to be 9.07 × 10^11^ particles/cm^2^ based on the XRD (according to the XRD results, the average diameter of the zirconia nanoparticles was 10.5 nm), as shown in Figure 2A. Using the laser spot size and the boundary density of the adsorbing molecules on the surface of the nanoparticles (~0.5 nmol/cm^2^), *N*_*Surf*_ can be calculated as 6.15 × 10^6^. From the observed spectra, the ratio of intensities I_Surf_/I_Bulk_ = 1765.42/5051.69 = 0.349 can be obtained. Substituting the obtained values for these variables into Equation 3, the EF value is calculated as 4.32 × 10^3^.

An enhancement (by a factor ~3) of the Bg and Ag ZrO_2_ phonon modes at 330 and 475 cm^−1^, respectively, was also observed. This enhancement is similar to the enhancement mechanism for the normal mode of the molecule. The results show that an EF of more than 10^3^ is achieved for 4-MBA on ZrO_2_ nanoparticles. It has not previously been reported that SERS signals are observed on the surface of ZrO_2_, but the results are similar to the EF for other semiconductor nanoparticles that have been reported previously.

### CT Mechanism

#### a1 and b2 Modes of 4-MBA

**Figure 4:**
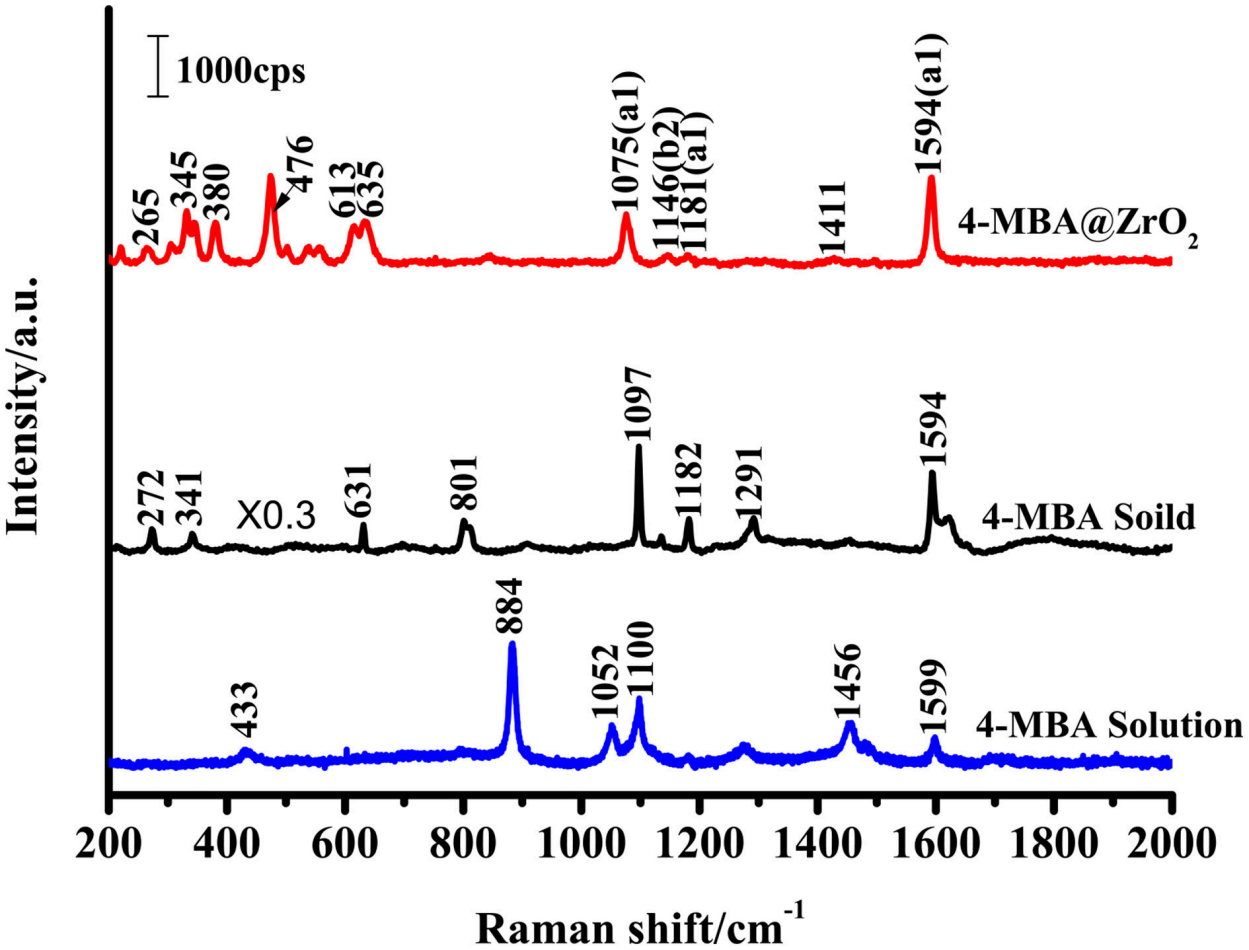
Raman spectra of 4-MBA adsorbed on ZrO_2_ nanoparticles, 4-MBA solid, and 4-MBA solution (0.1 mol/L in ethanol) with 532 nm excitations.

The SERS signal of a near-monolayer of 4-MBA adsorbed on the surface of ZrO_2_ nanoparticles was observed (the excess of 4-MBA on the surface was washed away with a solvent). Figure 4 shows the Raman spectra for the bulk 4-MBA and a solution dissolved in ethanol (0.1 mol/L), as well as the enhanced Raman spectrum of 4-MBA on a ZrO_2_ substrate. The only difference between the conventional Raman spectroscopy and SERS samples is the amount of 4-MBA molecules: in the former, a large number of pure 4-MBA molecules are present, while the latter utilizes a monolayer of 4-MBA molecules present only on the ZrO_2_ substrate. All of the other test conditions and procedures are the same. A distinct 4-MBA signal was observed on the ZrO_2_ substrate with clear Raman peaks at 1075, 1146, 1181, and 1594 cm^−1^. However, because SERS modifies the surface selection rules, there is a significant difference in the relative intensities of the peaks observed for the 4-MBA molecule in the Raman and SERS spectra. The bulk 4-MBA spectrum is mainly dominated by the fully symmetric a1 vibration mode, while the SERS spectrum for 4-MBA exhibits a strong contribution of the asymmetric b2 mode (1146 cm^−1^).

The Raman peak at 1075 cm^−1^ is attributed to aromatic ring vibration with a C-S stretching mode (ν12a, a1), while at approximately 1,594 cm^−1^ the Raman peak is assigned to the aromatic ring breathing mode (ν8a, b2). As shown in Figure 4, the Raman spectrum for 4-MBA adsorbed on the surface of zirconia nanoparticles was strongly enhanced compared to the conventional Raman spectrum for 4-MBA, and the Raman shift was significantly different from that of the bulk Raman spectrum. The spectrum of the 4-MBA adsorbed on the surface of zirconia nanoparticles exhibits a weak peak at 1146 cm^−1^ (ν15, b2) in the SERS spectrum (attributed to the C-H deformation vibrational mode), which is generally considered to be due to the Herzberg-Teller contribution that produces enhanced CT effects. The SERS spectrum of 4-MBA on the surface of ZrO_2_ is almost identical to that observed on the surface of ZrO_2_ and TiO_2_ in the literature. We also calculated the value of P_CT_, which was calculated through the expression which was defined as “P_CT_ = R/(1 + R)” for the degree of charge transfer(R = I_b2_/I_a1_). As shown in Figure S4, the P_CT_ of 4-MBA is near 0.5 at the excitation wavelengths of 532, 633, and 785 nm. It was observed that the degree of charge transfer increases with the increase of laser energy.

#### Direction of CT Effects

There are two main ways to explain the enhancement mechanism of the SERS effect: chemical mechanisms (CT) and electromagnetic mechanisms (EM). Chemical enhancement (CE) is a resonance-like Raman process. It is generated by chemical adsorption interactions between the substrate and the adsorbed molecules and photoinduced charge transfer, resulting in an increase in the polarizability and an increase in the distribution number of the adsorbed molecules in the excited state. The electromagnetic mechanisms (EM) is caused by an increase in the local electric field induced by the laser on the surface of the nanoparticles (Kadkhodazadeh et al., 2014; Mogensen and Kneipp, 2014). According to the literature, for most semiconductor materials, the surface plasmon resonance frequency is in the infrared region, away from the excitation wavelength (532 nm) used to obtain the SERS phenomenon in this study (Wang et al., 2009; Xue et al., 2012). Therefore, the SERS enhancement of ZrO_2_ in this work does not include EM contributions, but mainly contributes to CE contributions. In Figure 4, the SERS spectrum of 4-MBA exhibit a non-totally symmetric b2 mode at 1146 cm^−1^. As the CT mechanism is a resonance Raman-like process, the charge transfers occurred between the ZrO_2_ nanocrystals and 4-MBA molecules. When the organic molecules and semiconductor are in contact with each other, a reasonable charge distribution will exist around the interface within a very short time. A new electronic state then forms at the interface.

**Figure 5:**
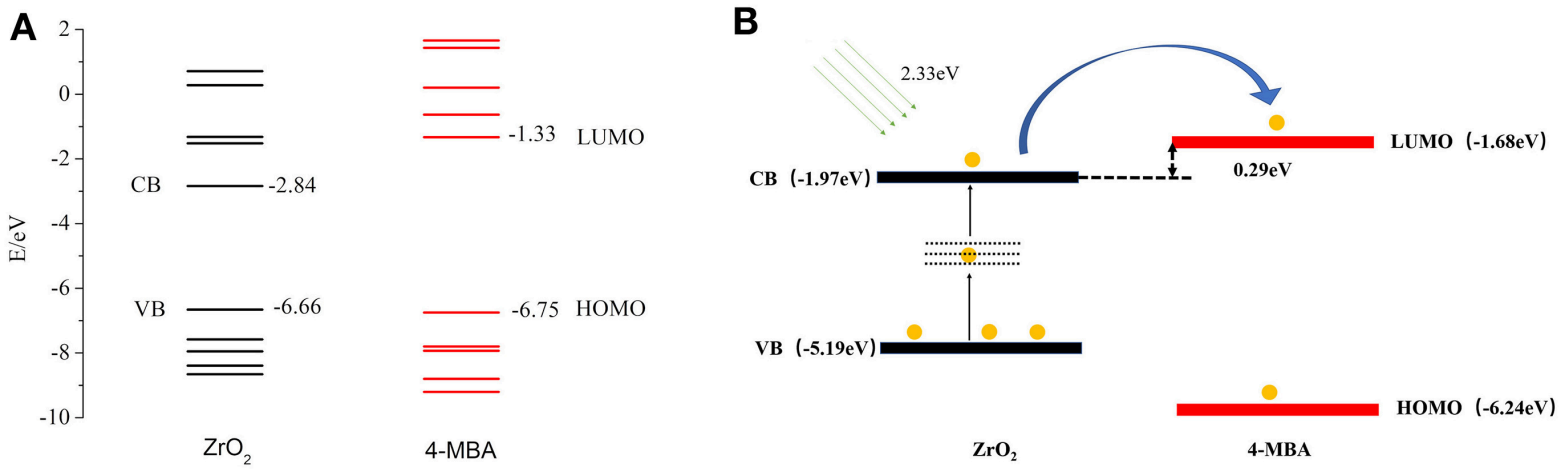
**(A)** The molecular orbital illustrations of the 4-MBA molecule and ZrO_2_ nanoparticles from calculations by Gaussian 09. **(B)** The schematic energy-level diagram at a ZrO_2_/4-MBA molecule interfaces, and also the position of the HOMO and the LUMO of 4-MBA and the position of CB and VB of ZrO_2_ nanoparticles.

To determine the direction of charge transfer in the 4-MBA–ZrO_2_ complex, Gaussian 09 was used to calculate the highest occupied molecular orbital (HOMO) and lowest unoccupied molecular orbital (LUMO) of 4-MBA molecules adsorbed on ZrO_2_ nanoparticles, as shown in Figure 5A. In the 4-MBA-modified ZrO_2_ system, the HOMO and LUMO for the 4-MBA occur at −6.75 and −1.33 eV, respectively. Thus, the CT between ZrO_2_ and the adsorbed 4-MBA molecule may occur as shown in Figure 5B. In these experiments, when irradiated under a 532 nm (ca. 2.33 eV) laser, the excited electrons of the ZrO_2_ conduction band (CB) migrate to the LUMO of the adsorbed molecules. This is a reasonable CT model to explain the SERS of ZrO_2_ modified by 4-MBA.

Zhao et al. reported the use of UV-Vis diffuse reflectance spectroscopy (DRS; Figure S5) (Zhang X. et al., 2015) and ultraviolet photoelectron spectroscopy (UPS; Figure S6) to calculate the HOMO and LUMO of the substrate and probe molecules. This can be calculated with Equation 4:

(4) W=hν-ΔE

The value of hν is 21.22 eV, and thus the valence band (VB) of ZrO_2_ is calculated to occur at −5.19 eV. The band gap of ZrO_2_ is 4.95 eV, and the CB of ZrO_2_ is situated at −1.97 eV. As shown in Figure 5B, in the ZrO_2_ system modified by 4-MBA, the HOMO of the 4-MBA is at −6.24 eV and the LUMO is at −1.68 eV. Therefore, considering the relative band structure of the ZrO_2_ system with 4-MBA, the CT direction between the ZrO_2_ and 4-MBA determined by the theoretical calculations and UPS results is consistent, i.e., the CT occurs from the zirconium dioxide to the 4-MBA molecules.

#### SERS Spectra of the ZrO_2_ Surface Modified by 4-MBA, 4-MPY, and PATP

**Figure 6:**
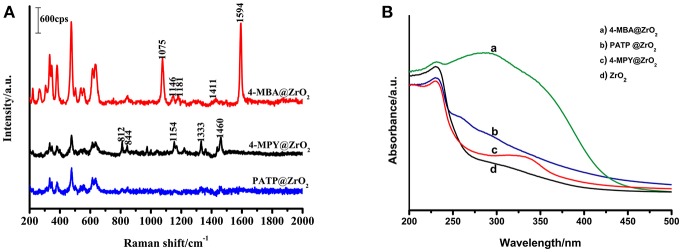
**(A)** SERS spectra of 4-MBA, PATP, 4-MPY adsorbed on ZrO_2_ nanoparticles; **(B)** UV-vis spectra of ZrO_2_ nanoparticles and 4-MBA, PATP, 4-MPY adsorbed on ZrO_2_ nanoparticles.

To illuminate that the enhancement mechanism of a ZrO_2_ surface modified by 4-MBA is the CT mechanism, the following experiment was performed. The surface of ZrO_2_ nanoparticles were modified with different thiophenol-related molecules (4-MPY and PATP), as shown in Figure 6A. In addition, these thiophenol-based molecules possess functional groups on the benzene ring, such as C-COOH, N, or C-NH_2_. The electron attracting ability of these functional groups on the benzene ring occurs in the order of C-COOH (4-MBA) > N (4-MPY) > C-NH_2_ (PATP). Figure 6A shows the SERS spectra for 4-MBA, 4-MPY, and PATP adsorbed on ZrO_2_ nanoparticles. The results clearly show that the intensities decrease in the order of 4-MBA > 4-MPY > PATP. This is in agreement with the previous estimations.

As the probe molecules have almost the same Raman cross-sections, the experimental results described above are not due to different Raman cross-sections of the molecules. The SERS spectra effect is consistent with the ability of the mercapto-pair to attract electrons. These results indicate a correlation between the SERS signal and the ZrO_2_-to-molecula CT. This suggests that for ZrO_2_ nanoparticles surface-modified by other molecules, the primary enhancement mechanism is the CT mechanism. The stronger the electron-withdrawing groups in the molecules bonded on the surface of ZrO_2_, the greater the ability of ZrO_2_ for molecular CT will be, and a stronger SERS signal on the ZrO_2_ will be observed.

Comparisons of the optical properties of ZrO_2_ nanoparticles modified by molecules with those of pure ZrO_2_ were carried out by using UV-Vis spectroscopy (Figure 6B). It is clear that the adsorption of organic molecules has a significant influence on the absorption spectrum of ZrO_2_. It was observed that the typical ZrO_2_ direct band gap adsorbs organic molecules with different degrees of narrowing and widening.

Figure S5 shows the spectra of ZrO_2_ nanoparticles surface-modified by 4-MBA, 4-MPY, and PATP, as well as pure ZrO_2_ nanoparticles monitored with room temperature UV-vis DRS. Using the Kubelka–Munk function, the bandgap energy of the pure ZrO_2_ and molecule-modified ZrO_2_ were calculated based on the photon energy [(αE)^2^] function. The resulting band energy is 4.95 eV (250 nm) for the pure ZrO_2_ nanoparticles, 3.22 eV (385 nm) for the 4-MBA-modified ZrO_2_, 4.88 eV (254 nm) for the 4-MPY-modified ZrO_2_, and 4.91 eV (253 nm) for the PATP-modified ZrO_2_. The interaction between the adsorbed molecules and the ZrO_2_ nanoparticle substrate resulted in these shifts. The transformation may be due to the interaction of the nanoparticles with probe molecules. The observed shift shows that the bandgap of the 4-MBA–ZrO_2_ system has a lower optical transition energy than that of pure ZrO_2_ due to the CT transition. The 4-MBA–ZrO_2_ system has the lowest optical transition energy, which explains why the ZrO_2_ modified by 4-MBA has the strongest SERS effect.

## Conclusion

This study first observed that the SERS spectrum can be obtained on the surface of ZrO_2_. The results show that the SERS spectrum of 4-MBA on ZrO_2_ is mainly due to the contribution of the CT mechanism, and the intrinsic properties of the adsorbed molecules and the surface properties of the semiconductor have a significant effect on SERS. The plentiful surface state of the ZrO_2_ active substrate is beneficial for the occurrence of CT resonance between molecules and the ZrO_2_ to produce a SERS effect. The results show that the ZrO_2_ nanoparticles prepared through the sol-gel method at a calcination temperature of 500°C exhibit the strongest SERS effect on the surface-adsorbed probe molecules. This study demonstrates for the first time that ZrO_2_ can be used as a Raman enhancement substrate material, breaking through the limitations of the precious metal substrates used in traditional SERS technology, and further broadening the application of semiconductor oxides as substrate materials for SERS detection. Also, the SERS study on the size effect of nano-sized ZrO_2_ is expected to promote the better performance of nano-ceramic materials to enable the emergence of new properties and functions.

## Author Contributions

XiuS designed the experiment. PJ conducted experiments and characterization. ZW funded some of the subject experiments. XiaS assisted in the theoretical simulation. YZ and YL participated in the discussion. PJ, XiuS, and ZM wrote the manuscript together.

### Conflict of Interest Statement

The authors declare that the research was conducted in the absence of any commercial or financial relationships that could be construed as a potential conflict of interest.

## References

[B1] Alessandri I. Lombardi J. R. (2016). Enhanced raman scattering with dielectrics. Chem. Rev. 116, 14921–14981. 10.1021/acs.chemrev.6b00365 27739670

[B2] Becker J. Hald P. Bremholm M. Pedersen J. S. Chevallier J. Iversen S. B. (2008). Critical size of crystalline ZrO2 nanoparticles synthesized in near- and supercritical water and supercritical isopropyl alcohol. ACSnano 2, 1058–1068. 10.1021/nn7002426 19206504

[B3] Carlone C. (1992). Raman spectrum of zirconia-hafnia mixed crystals. Phys. Rev. B 45, 2079–2084. 10.1103/physrevb.45.2079 10001722

[B4] Chang S. Doong R. (2007). Interband transitions in Sol–Gel-Derived ZrO_2_ films under different calcination conditions. Chem. Mater. 19, 4804–4810. 10.1021/cm070606n

[B5] De Aza A. H. Chevalier J. Fantozzi G. Schehl M. Torrecillas R. (2002). Crack growth resistance of alumina, zirconia andzirconia toughened alumina ceramics for joint prostheses. Biomaterials 23, 937–945. 10.1016/S0142-9612(01)00206-X 11774853

[B6] Duan H. Unno M. Yamada Y. Sato S. (2017). Adsorptive interaction between 1,5-pentanediol and Mgo-Modified ZrO_2_ catalyst in the vapor-phase dehydration to produce 4-Penten-1-Ol. Appl. Cataly. A Gen. 546, 96–102. 10.1016/j.apcata.2017.07.048

[B7] Fu X. Wang Y. Liu Y. Liu H. Fu L. Wen J.. (2019). A graphene oxide/gold nanoparticle-based amplification method for SERS immunoassay of cardiac troponin I. Analyst 144, 1582–1589. 10.1039/c8an02022a 30666995

[B8] Gan X. Yu Z. Yuan K. Xu C. Wang X. Zhu L. (2017). Preparation of a CeO_2_-nanoparticle thermal radiation shield coating on ZrO_2_ fibers Via a hydrothermal method. Ceram. Int. 43, 14183–14191. 10.1016/j.ceramint.2017.07.161

[B9] Han X. X. Ji W. Zhao B. Ozaki Y. (2017). Semiconductor-enhanced raman scattering: active nanomaterials and applications. Nanoscale 9, 4847–4861. 10.1039/c6nr08693d 28150834

[B10] Heuer A. H. Claussen N. Kriven W. M. Ruhle M. (1982). Stability of tetragonal ZrO2 particles in ceramic matrices. J. Am. Ceram. Soc. 65, 643–650. 10.1111/j.1151-2916.1982.tb09946.x

[B11] Huang Y. F. Zhang M. Zhao L. B. Feng J. M. Wu D. Y. Ren B.. (2014). Activation of oxygen on gold and silver nanoparticles assisted by surface plasmon resonances. Angew. Chem. Int. Edn. 53, 2353–2357. 10.1002/ange.201310097 24481674

[B12] Jiang L. You T. Yin P. Shang Y. Zhang D. Guo L. (2013). Surface-enhanced raman scattering spectra of adsorbates on Cu(2)O nanospheres: charge-transfer and electromagnetic enhancement. Nanoscale 5, 2784–2789. 10.1039/c3nr33502j 23435689

[B13] Kadkhodazadeh S. de Lasson J. R. Beleggia M. Kneipp H. Wagner J. B. Kneipp K. (2014). Scaling of the surface plasmon resonance in gold and silver dimers probed by eels. J. Phys. Chem. C. 118, 5478–5485. 10.1021/jp500288s

[B14] Kralik B. Chang E. K. Louie S. G. (1998). Structural properties and quasiparticle band structure of zirconia. Phys. Rev. B. 57:7027 10.1103/physrevb.57.7027

[B15] Li J. Chen L. Lou T. Wang Y. (2011). Highly sensitive SERS detection of As3+ ions in aqueous media using glutathione functionalized silver nanoparticles. ACS Appl. Mater. Interfaces 3, 3936–3941. 10.1021/am200810x 21916441

[B16] Li X. Xu Y. Mao X. Zhu Q. Xie J. Feng M. (2017). Investigation of optical, mechanical, and thermal properties of ZrO_2_-Doped Y_2_O_3_ transparent ceramics fabricated by Hip. Ceram. Int. 44, 1362–1369. 10.1016/j.ceramint.2017.08.204

[B17] Lombardi J. R. Birke R. L. (2014). Theory of surface-enhanced raman scattering in semiconductors. J. Phys. Chem. C 118, 11120–11130. 10.1021/jp5020675

[B18] Long D. Niu M. Tan L. Fu C. Ren X. Xu K.. (2017). Ball-in-Ball ZrO_2_ nanostructure for simultaneous Ct imaging and highly efficient synergic microwave ablation and tri-stimuli-responsive chemotherapy of tumors. Nanoscale 9, 8834–8847. 10.1039/c7nr02511d 28632268

[B19] Mogensen K. B. Kneipp K. (2014). Size-dependent shifts of plasmon resonance in silver nanoparticle films using controlled dissolution: monitoring the onset of surface screening effects. J. Phys. Chem. C 118, 28075–28083. 10.1021/jp505632n

[B20] Navio J. Hidalgo M. Colon G. Botta S. Litter M. (2001). Preparation and physicochemical properties of ZrO_2_ and Fe/ZrO_2_ prepared by a Sol– Gel Technique. Langmuir 17, 202–210. 10.1021/la000897d

[B21] Scott B. L. Carron K. T. (2016). Dynamic raman scattering studies of coated gold nanoparticles: 4-mercaptopyridine, 4-mercaptophenol, and benzenethiol. J. Phys. Chem. C 120, 20905–20913. 10.1021/acs.jpcc.6b02617

[B22] Shen W. Lin X. Jiang C. Li C. Lin H. Huang J.. (2015). Reliable quantitative sers analysis facilitated by core–shell nanoparticles with embedded internal standards. Angew. Chem. Int. Edn. 54, 7308–7312. 10.1002/ange.201502171 25939998

[B23] Shiohara A. Wang Y. Liz-Marzán L. M. (2014). Recent approaches toward creation of hot spots for sers detection. J. Photochem. Photobiol. C. 21, 2–25. 10.1016/j.jphotochemrev.2014.09.00

[B24] Sun Z. Zhao B. Lombardi J. R. (2007). ZnO nanoparticle size-dependent excitation of surface raman signal from adsorbed molecules: observation of a charge-transfer resonance. Appl. Phys. Lett. 91:221106 10.1063/1.2817529

[B25] Wang C. Guan Q. Wu F. Wang H. (2019). A novel Mgo doped Zr0.92y0.08O_2_-Î'(8ysz) with NaCl/KCl composite electrolyte for intermediate temperature fuel cells. Ceram. Int. 45, 1605–1608. 10.1016/j.ceramint.2018.10.035

[B26] Wang Y. Chen L. (2011). Quantum dots, lighting up the research and development of nanomedicine. Nanomedicine 7, 385–402. 10.1016/j.nano.2010.12.006 21215327

[B27] Wang Y. Ji W. Sui H. Kitahama Y. Ruan W. Ozaki Y. (2014). Exploring the effect of intermolecular H-bonding: a study on charge-transfer contribution to surface-enhanced raman scattering of P-mercaptobenzoic acid. J. Phys. Chem. C 118, 10191–10197. 10.1021/jp5025284

[B28] Wang Y. Ruan W. Zhang J. Yang B. Xu W. Zhao B. (2009). Direct observation of surface-enhanced Raman scattering in ZnO nanocrystals. J. Raman Spectrosc. 40, 1072–1077. 10.1002/jrs.2241

[B29] Wang Y. Sun Z. Wang Y. Hu H. Zhao B. Xu W.. (2007). Surface-enhanced raman scattering on mercaptopyridine-capped CdS microclusters. Spectrochim. Acta. Part A Mole. Biomol. Spectrosc. 66, 1199–1203. 10.1016/j.saa.2006.06.008 16920002

[B30] Wang Y. Wang Y. Wang W. Sun K. Chen L. (2016). Reporter-embedded SERS tags from gold nanorod seeds: selective immobilization of reporter molecules at the tip of nanorods. ACS Appl. Mater. Interfaces 8, 28105–28115. 10.1021/acsami.6b04216 27696805

[B31] Wang Y. Yan B. Chen L. (2013). SERS tags: novel optical nanoprobes for bioanalysis. Chem. Rev. 113, 1391–1428. 10.1021/cr300120g 23273312

[B32] Xu S. Xu Y. Liu Y. Fang M. Wu X. Min X. (2017). Fabrication and abrasive wear behavior of ZrO_2_-SiC-Al_2_O_3_ ceramic. Ceram. Int. 43, 15060–15067. 10.1016/j.ceramint.2017.08.032

[B33] Xue X. Ji W. Mao Z. Mao H. Wang Y. Wang X. (2012). Raman investigation of nanosized TiO2: effect of crystallite size and quantum confinement. J. Phys. Chem. C 116, 8792–8797. 10.1021/jp2122196

[B34] Yang L. Jiang X. Ruan W. Zhao B. Xu W. Lombardi J. R. (2008). Observation of enhanced raman scattering for molecules adsorbed on Tio_2_ nanoparticles: charge-transfer contribution. J. Phys. Chem. C 112, 20095–20098. 10.1021/jp8074145

[B35] Yin P. G. Jiang L. You T. T. Zhou W. Li L. Guo L. (2010). Surface-enhanced Raman spectroscopy with self-assembled cobalt nanoparticle chains: comparison of theory and experiment. Phys. Chem. 12, 10781–10785. 10.1039/c002662j 20657894

[B36] Zhang L. Huang Z. Pan W. Krell A. (2015). High transparency Nd: Y2o3ceramics prepared with La_2_O_3_ and ZrO_2_ additives. J. Am. Ceram. Soc. 98, 824–828. 10.1111/jace.13354

[B37] Zhang X. Yu Z. Ji W. Sui H. Cong Q. Wang X. (2015). Charge-transfer effect on Surface-Enhanced Raman Scattering (SERS) in an ordered Ag Nps/4-mercaptobenzoic Acid/TiO_2_ system. J. Phys. Chem. C 119, 22439–22444. 10.1021/acs.jpcc.5b06001

[B38] Zhao L. B. Zhang M. Huang Y. F. Williams C. T. Wu D. Y. Ren B.. (2014). Theoretical study of plasmon-enhanced surface catalytic coupling reactions of aromatic amines and nitro compounds. J. Phys. Chem. Lett. 5, 1259–1266. 10.1021/jz5003346 26274481

[B39] Zhao X. Vanderbilt D. (2002). Phonons and lattice dielectric properties of zirconia. Phys. Rev. B. 65:075105 10.1103/PhysRevB.65.075105

[B40] Zheng H. Liu K. Cao H. Zhang X. (2009). L-Lysine-assisted synthesis of ZrO_2_ nanocrystals and their application in photocatalysis. J. Phys. Chem. C 113, 18259–18263. 10.1021/jp9057324

[B41] Zhou L. Xu J. Li X. Wang F. (2006). Metal oxide nanoparticles from inorganic sources via a simple and general method. Mater. Chem. Phys. 97, 137–142. 10.1016/j.matchemphys.2005.07.062

