# Supplementary material for: Direct Observation of Enhanced Raman Scattering on Nano-Sized ZrO2 Substrate: Charge-Transfer Contribution
Source: Front Chem. 2019 Apr 17;7:245. doi: 10.3389/fchem.2019.00245 (PMC6478807; doi:10.3389/fchem.2019.00245)
Supplement: Supplementary file 1 [file Data_Sheet_1.docx]

Supplementary Material

**
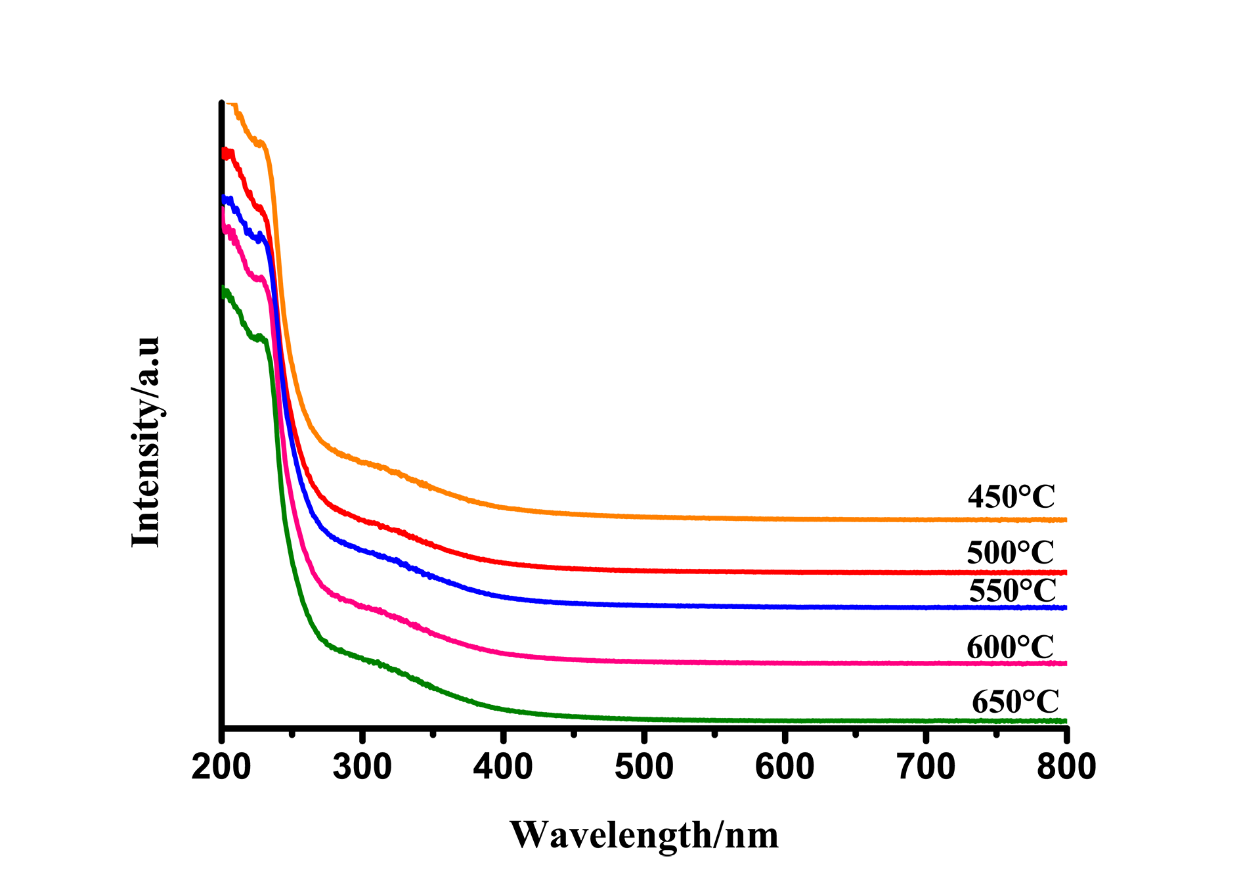
**

**Figure S1**. UV-vis spectra of ZrO_2_ nanoparticles with different calcination temperature.


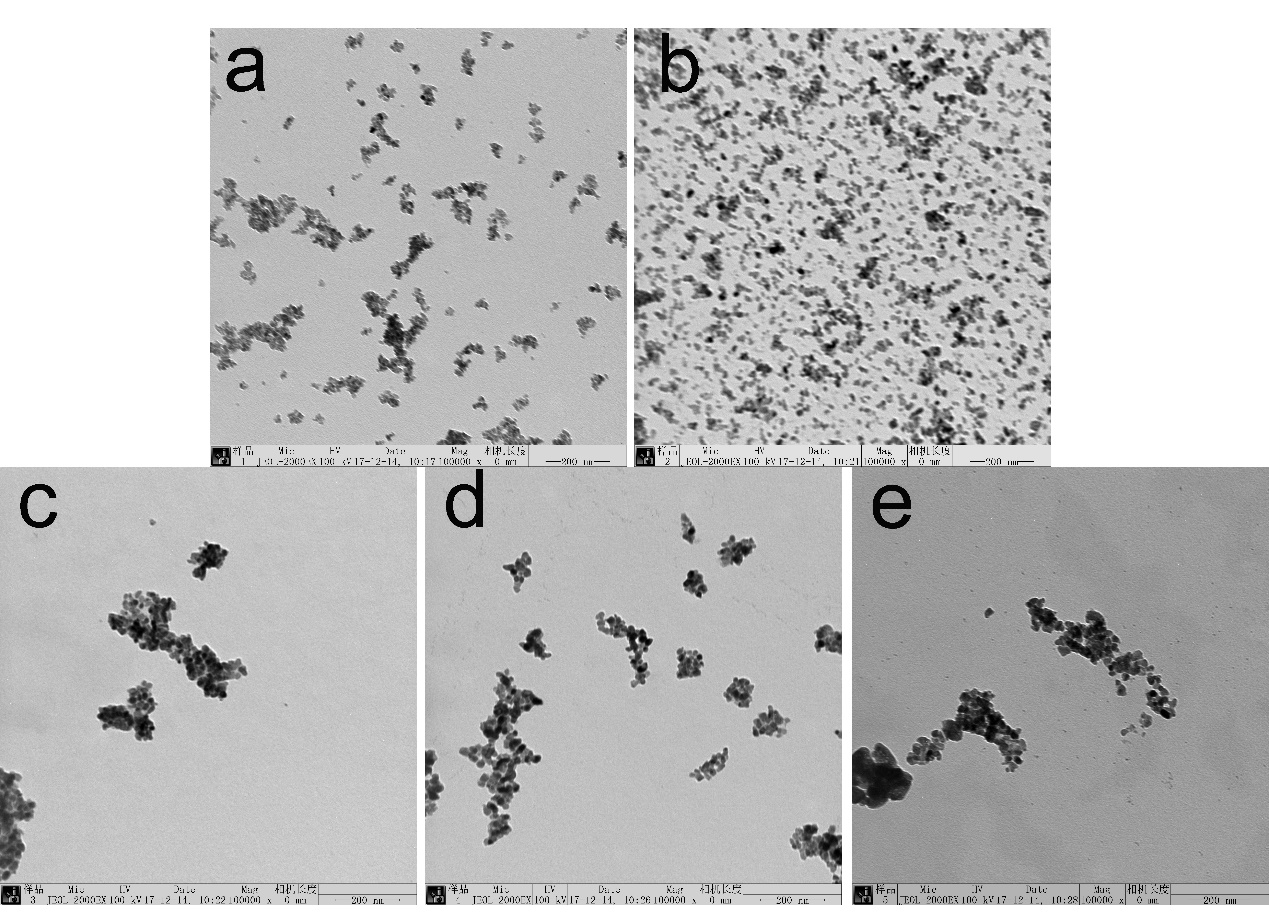


**Figure S2**. Transmission electron microscopy (TEM) images of the ZrO_2_ nanoparticles calcined at (a)450℃, (b)500 ℃, (c)550℃, (d)600℃, and (e)650℃.


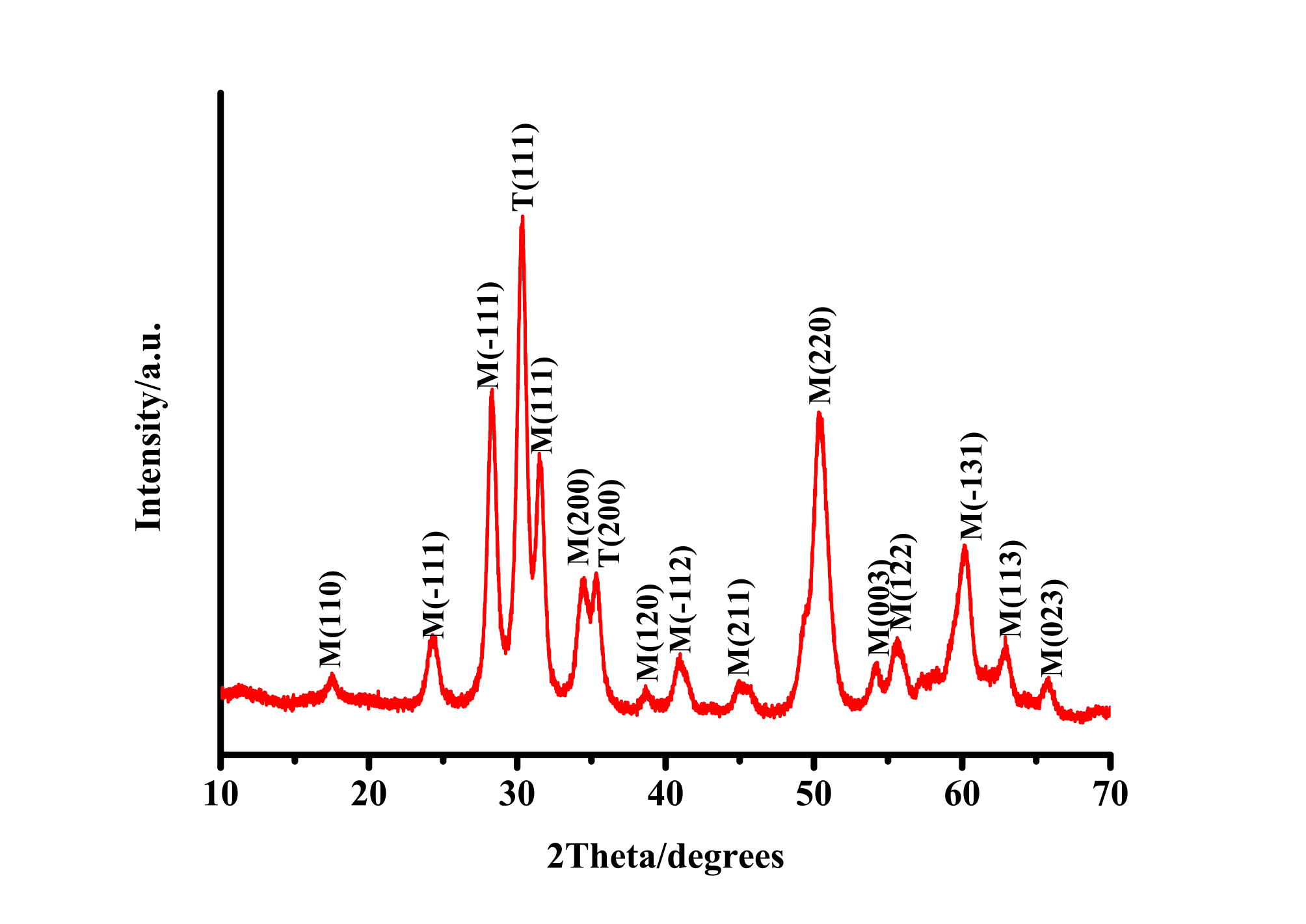


**Figure S3**. XRD patterns of ZrO_2_ nanoparticles calcined at 500℃. Peaks corresponding to monoclinic phase ZrO_2_ are denoted with a “M”. the peak corresponding to tetragonal phase ZrO_2_ is represented by “T”.

**
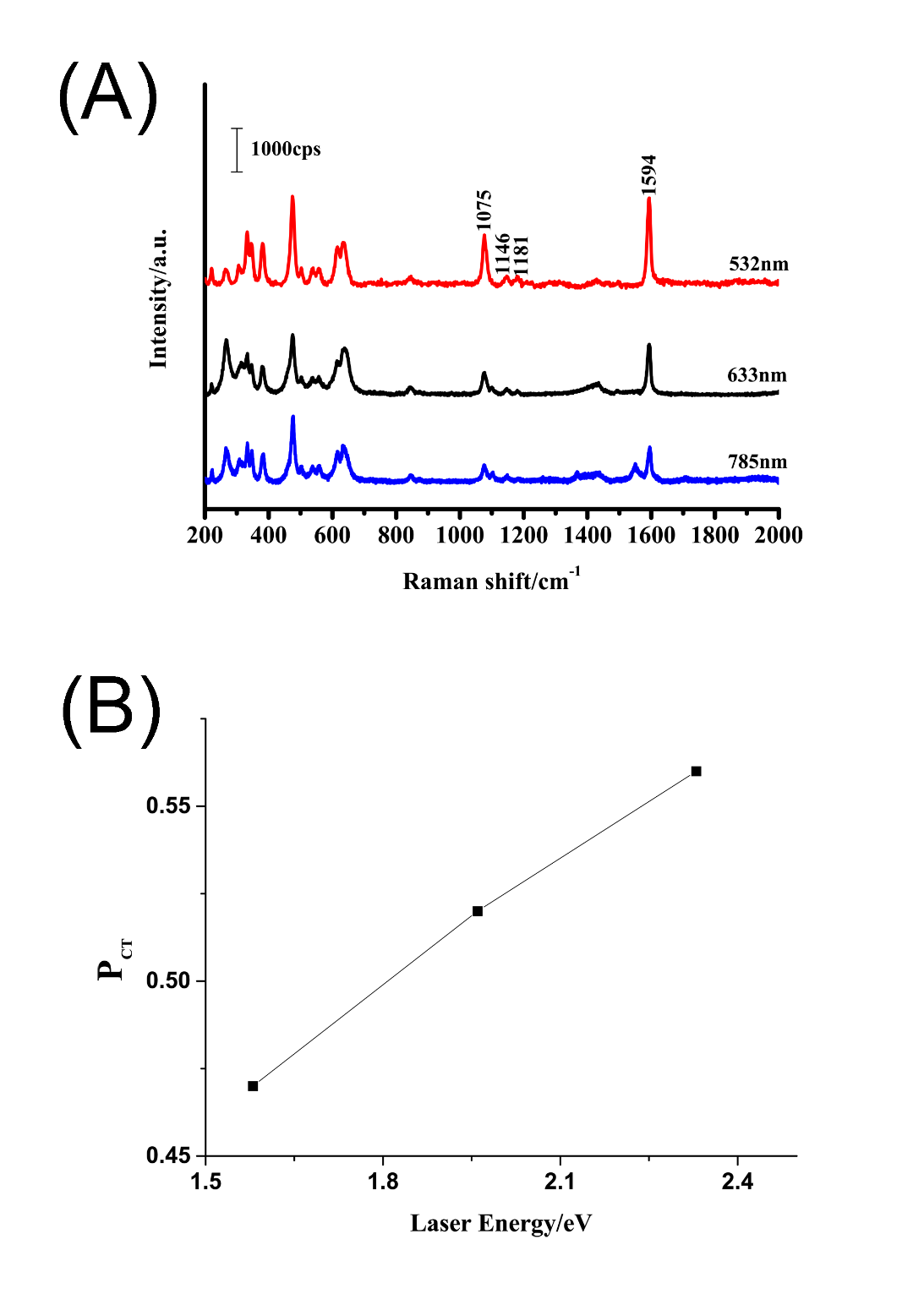
**

**Figure S4.** Degree of charge-transfer (P_CT_) as a function of excitation wavelengths of the SERS of 4-MBA on ZrO_2_ at the excitation wavelengths of 532, 633, and 785 nm.


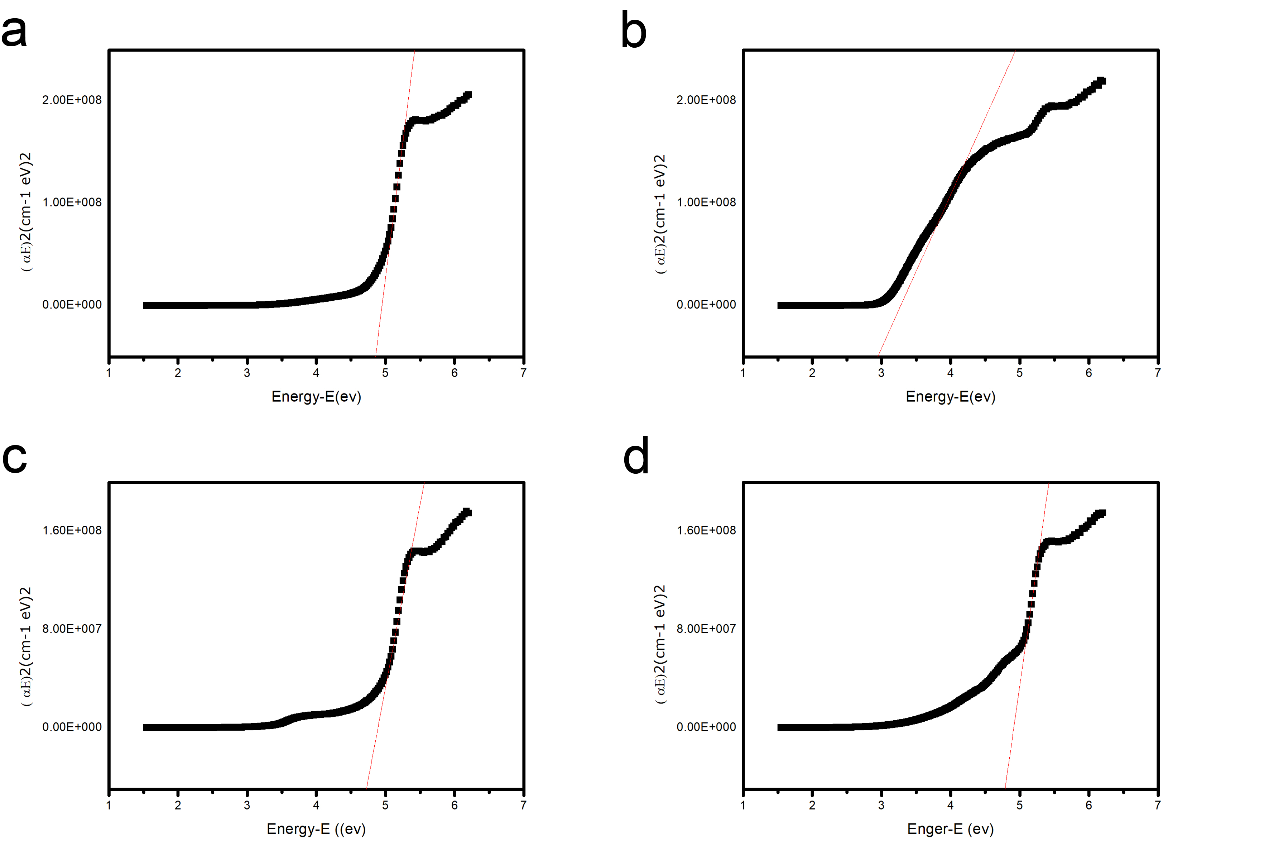


**Figure S5.** UV-vis DRS spectra of (a) pure ZrO_2_ nanoparticle, and ZrO_2_ nanoparticle surface-modified by (b)4-MBA, (c)4-MPY, (d)PATP and monitored at room temperature.

**Table S1.** Raman Frequencies and Assignments of 4-Mercaptobenzoic acid(4-MBA) in the 800-2000 cm^-1^ Region^a^

| assignment | symmetry | 4-MBA onSnO_2_ | 4-MBA onTiO_2_ | 4-MBA on ZnO | 4-MBA Bulk  (this work) | 4-MBA Solution  (this work) | 4-MBA on ZrO_2_  (this work） |
| --- | --- | --- | --- | --- | --- | --- | --- |
| νCC aromatic ring characteristic vibrations | *ν*8a (a1) | 1078 | 1078 | 1087 | 1096 | 1100 | 1075 |
| νCH the C-H deformation modes | (ν15, b2) | 1145 | 1148 | 1150 |  |  | 1146 |
| νCH the C-H deformation modes | (ν9 a1) | 1184 | 1182 |  | 1180 | 1181 | 1181 |
|  | *ν*(COO^-^) | 1396 | 1414 | 1414 |  | 1456 | 1411 |
| νCC aromatic ring characteristic vibrations | *ν*12 (a1) | 1594 | 1594 | 1593 | 1954 | 1599 | 1594 |

^a^Assignments and band positions (cm^-1^) reported in previous articles (see refs 1-4) compared with those obtained in this work for the 4-MBA on ZrO2.

1.Sun, Z.; Zhao, B.; Lombardi, J. R., ZnO nanoparticle size-dependent excitation of surface Raman signal from adsorbed molecules: Observation of a charge-transfer resonance. *Applied Physics Letters* **2007,** *91* (22), 221106.

2.Jiang, L.; Yin, P.; You, T.; Wang, H.; Lang, X.; Guo, L.; Yang, S., Highly reproducible surface-enhanced Raman spectra on semiconductor SnO_2_ octahedral nanoparticles. *Chemphyschem: a European journal of chemical physics and physical chemistry* **2012,** *13* (17), 3932-6.

3.Yang, L.; Jiang, X.; Ruan, W.; Zhao, B.; Xu, W.; Lombardi, J. R., Observation of enhanced Raman scattering for molecules adsorbed on TiO_2_ nanoparticles: charge-transfer contribution. *The Journal of Physical Chemistry C* **2008,** *112* (50), 20095-20098.

4.Zhihua Sun, Bing Zhao, and John R. Lombardi, ZnO nanoparticle size-dependent excitation of surface Raman signal from adsorbed molecules: Observation of a charge-transfer resonance. Appl. Phys. Lett. 91, 221106 (2007);


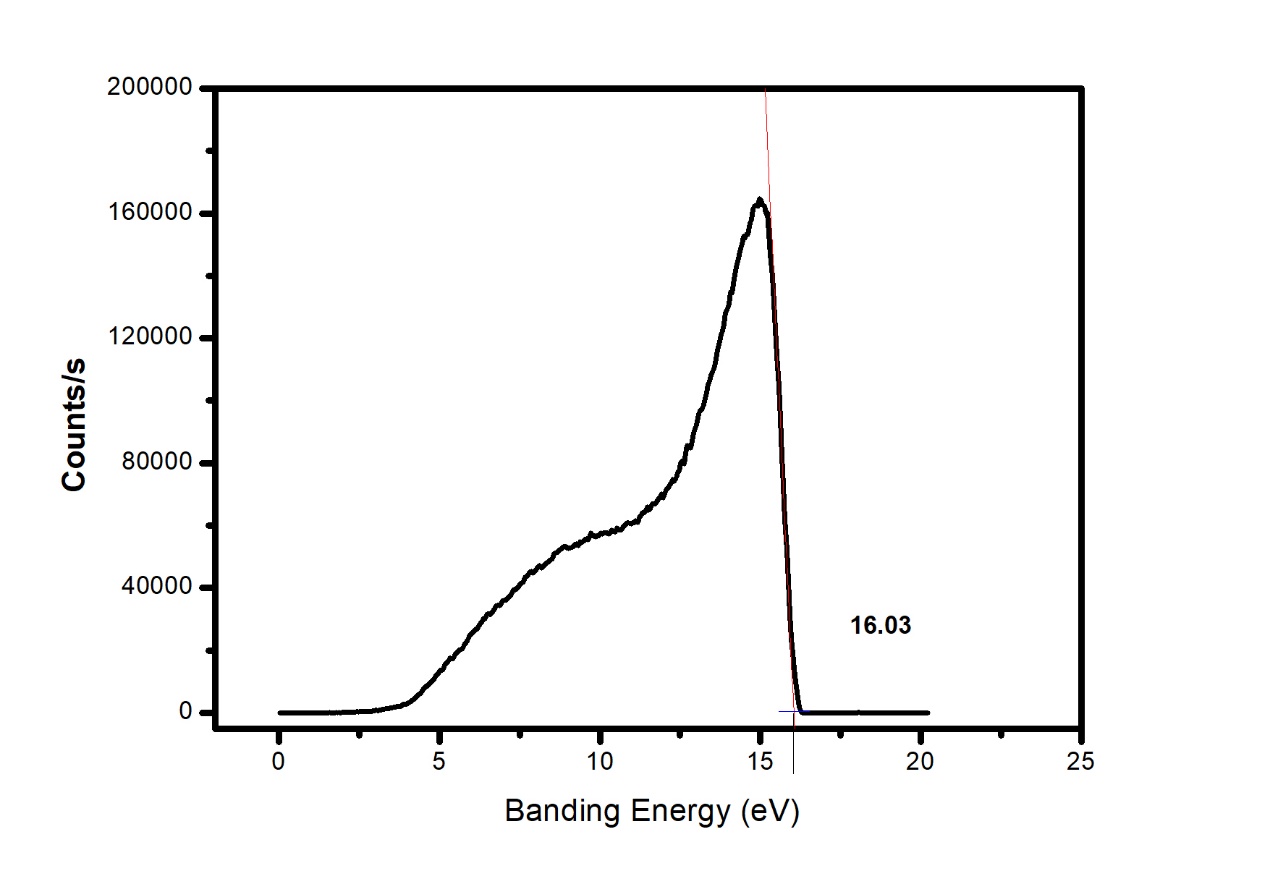


**Figure S6**. The ultraviolet photoelectron spectroscopy (UPS) of 4-MBA adsorbed on ZrO_2_ nanoparticles.
